# Cardiotocography analysis by empirical dynamic modeling and Gaussian processes

**DOI:** 10.3389/fbioe.2022.1057807

**Published:** 2023-01-12

**Authors:** Guanchao Feng, Cassandra Heiselman, J. Gerald Quirk, Petar M. Djurić

**Affiliations:** ^1^ Department of Electrical and Computer Engineering, Stony Brook University, Stony Brook, NY, United States; ^2^ Department of Obstetrics and Gynecology, Renaissance School of Medicine, Stony Brook University, Stony Brook, NY, United States

**Keywords:** attractor manifold, cardiotocography, empirical dynamic modelling, fetal heart rate, uterine activity

## Abstract

**Introduction:** During labor, fetal heart rate (FHR) and uterine activity (UA) can be continuously monitored using Cardiotocography (CTG). This is the most widely adopted approach for electronic fetal monitoring in hospitals. Both FHR and UA recordings are evaluated by obstetricians for assessing fetal well-being. Due to the complex and noisy nature of these recordings, the evaluation by obstetricians suffers from high interobserver and intraobserver variability. Machine learning is a field that has seen unprecedented advances in the past two decades and many efforts have been made in computerized analysis of CTG using machine learning methods. However, in the literature, the focus is often only on FHR signals unlike in evaluations performed by obstetricians where the UA signals are also taken into account.

**Methods:** Machine learning is a field that has seen unprecedented advances in the past two decades and many efforts have been made in computerized analysis of CTG using machine learning methods. However, in the literature, the focus is often only on FHR signals unlike in evaluations performed by obstetricians where the UA signals are also taken into account. In this paper, we propose to model intrapartum CTG recordings from a dynamical system perspective using empirical dynamic modeling with Gaussian processes, which is a Bayesian nonparametric approach for estimation of functions.

**Results and Discussion:** In the context of our paper, Gaussian processes are capable for simultaneous estimation of the dimensionality of attractor manifolds and reconstructing of attractor manifolds from time series data. This capacity of Gaussian processes allows for revealing causal relationships between the studied time series. Experimental results on real CTG recordings show that FHR and UA signals are causally related. More importantly, this causal relationship and estimated attractor manifolds can be exploited for several important applications in computerized analysis of CTG recordings including estimating missing FHR samples, recovering burst errors in FHR tracings and characterizing the interactions between FHR and UA signals.

## 1 Introduction

**FIGURE 1:**
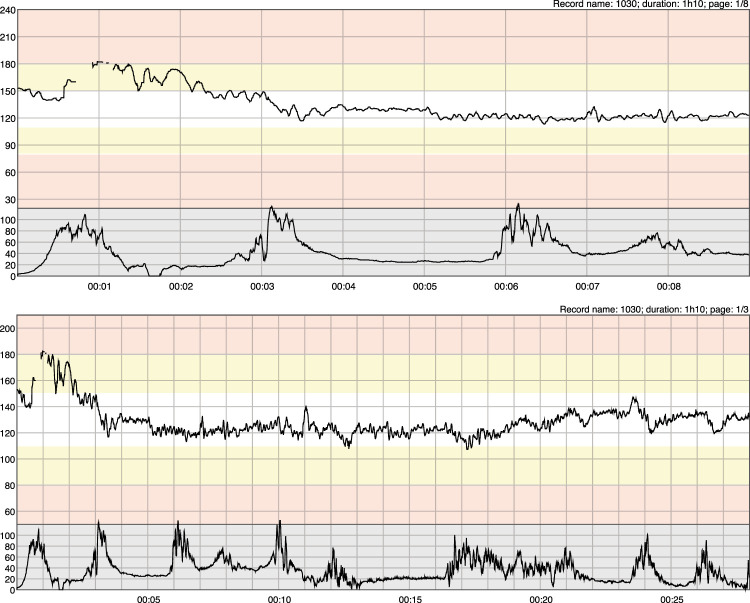
A segment of CTG recording from an open access intrapartum CTG database [described in (Chudáček et al., 2014)] in US (top two plots) and in EU (bottom two plots) paper formats, respectively.

During labor, without adequate level of oxygenation, a fetus can become hypoxic and acidotic. Very small changes in pH may significantly affect the function of various fetal organ systems, such as the central nervous system and the cardiovascular system (Omo-Aghoja, 2014). Oxygen deprivation or hypoxia is one of the most common challenges in fetal life, and can cause permanent brain damage or even death of the fetus (Giussani, 2016). Continuous electronic fetal monitoring (EFM) is commonly performed by Cardiotocography (CTG). The CTG monitor samples both fetal heart rate (FHR) and maternal uterine contractions or uterine activity (UA). The purpose of EFM is to alert obstetricians of these changes in fetal status for appropriate and timely intervention (Afors and Chandraharan, 2011). In Figure 1, we see the same CTG recording displayed in US format (horizontal: 3 cm/min, vertical: 30 bpm/cm) and in EU format (horizontal: 1 cm/min, vertical: 20 bpm/cm), respectively. In each plot, the upper tracing represents FHR signal and the lower tracing is the corresponding UA signal. Since the focus of our paper is on intrapartum CTG, in this work, the term CTG refers to intrapartum CTG if not specifically stated.

The CTG recordings are visually evaluated by experienced obstetricians, nurse-midwives and labor & delivery room nurses following clinical guidelines (Macones et al., 2008; Ayres-de Campos et al., 2015) that are based on FHR and interbeat variability, frequency and duration of uterine contractions, and the temporal relationship of decelerations of the FHR in relationship to the onset as well as the offset of uterine contractions. The FHR tracings are then usually categorized into one of three categories: category I as normal, category II as atypical or indeterminate, and category III as abnormal. Although category II patterns occur in the majority of fetuses in labor, there is still no standard approach to their management Clark et al. (2013).

After more than half a century of EFM in practice, its usefulness and benefits still remain controversial. During this period, there has been an increase in cesarean delivery and instrumental vaginal births (Alfirevic et al., 2006), and yet, the incidences of neonatal mortality and cerebral palsy have not been reduced (Bailey, 2009). However, it is worth noting that CTG has remained a widely used technology for assessing fetal wellbeing in real time during labor (Freeman et al., 2012; Nageotte, 2015). Presently, most hospitals in the United States offer CTG as the primary means of fetal surveillance during labor.

Several studies have reported that the evaluation of FHR tracings by obstetricians suffers from high interobserver and intraobserver variability. In a recent article, the agreement among expert obstetricians was investigated by having nine experienced obstetricians annotate 634 CTG recordings. Their results showed that the inter- and intra-observer variability was large and that the overall proportion of agreement among them only reached 48% (Hruban et al., 2015). There is little doubt that interpreting CTG recordings using morphological features is an exceptionally complex and often unsatisfactory exercise.

Computerized analysis of intrapartum CTG recordings is a logical approach because of its inherent “objectivity.” Computerized analysis has evolved from algorithms that literally implement the clinical guidelines to sophisticated machine learning techniques, which exploit patterns that cannot be discovered by human eyes. The interpretation of intrapartum CTG recordings, however, still remains challenging for computerized systems (Steer, 2014). Interestingly, none of the exciting breakthroughs in machine learning have contributed to revolutionizing the computerized analysis of intrapartum CTG recordings yet (Georgieva et al., 2019).

Unlike the assessment performed by obstetricians and physicians where FHR is evaluated jointly with UA and other clinical data, in the literature of automated analysis, the focus is often classification of FHR tracings (Georgoulas et al., 2017), and the analysis usually concentrates on the FHR signals only. That is, other intrapartum signals, such as UA and maternal heart rate (MHR), and clinical data are not considered. Since these other signals and data also provide valuable information about the fetal wellbeing, excluding them from the analysis is a disadvantage. Rare exceptions of articles where FHR signals are studied in conjunction with UA signals are (Romano et al., 2006; Cesarelli et al., 2010; Warmerdam et al., 2016; Warmerdam et al., 2018).

Empirical dynamic modeling (EDM) is a flexible data-driven framework for modeling non-linear dynamic systems. It is built upon the mathematical theory of reconstructing system attractors from time series data and is often used for studying systems with non-linear state-dependent behavior. An attractor of a system can be seen as a collection of states toward which the system tends to evolve under different initial conditions. Thus, reconstructing an attractors is of great importance in investigating system characteristics and behavior. A Gaussian process (GP) extends the concept of multivariate Gaussian distribution. The latter is defined for vectors of finite dimensions, whereas GPs are objects of infinite dimension, which gives them flexibility for modeling distributions of functions. Learning unknown functions (or mappings) lies at the core of solving many machine learning problems, and in practice, GPs provide a Bayesian non-parametric framework for learning unknown functions. Particularly, when we have to estimate an unknown function, we first specify a prior distribution for this function using a GP (instead of assuming an analytical form of it, like a polynomial of some form or a set of superimposed sinusoids). Then, we learn the posterior distribution of the function by incorporating the observed data and using Bayes’ theorem. We point out that our prior knowledge of the unknown function (e.g., periodicity) can be encoded in the prior distribution of the GP.

In this paper, we present our work on intrapartum CTG analysis using empirical dynamic modelling (EDM) with Gaussian processes. With our approach, we are able to reconstruct attractor manifolds from time series data within the Bayesian non-parametric probabilistic framework. Instead of only relying on FHR signals, both FHR and UA signals are taken into account from a dynamical system perspective. The Bayesian nature allows for data efficiency and proper quantification of uncertainties in learning.

The article is structured as follows. In the next section, we first briefly introduce an open access CTG database that has been widely adopted in computerized analysis of CTG recordings. We also selected this database for our experiments. Then we discuss the background and some fundamentals of EDM and GP. In Section 3, we describe our GP-based EDM in details. In Section 4 and Section 5 we present (direct and indirect) applications of GP-based EDM in CTG analysis. Finally, in Section 6, we conclude this article with some final remarks.

## 2 Background

### 2.1 Open access intrapartum CTG database

In this work, in all the experiments we used an open access intrapartum CTG database, known as CTU-UHB database. The intrapartum CTG database consists of a total of 552 intrapartum recordings, which were acquired between April 2010 and August 2012 at the Obstetrics Ward of the University Hospital in Brno (UHB), Czech Republic. The data were collected and anonymized at the UHB and de-identified at the Czech Technical University (CTU). The database is composed of a mixture of recordings acquired by ultrasound Doppler probes, direct scalp measurements or a combination of both. Each CTG record contains time information and signal of fetal heart rate and uterine contractions, both sampled at 4 Hz. When a signal was recorded using an internal scalp electrode, it also contained T/QRS ratio and information about biphasic T-waves. All recordings have available biochemical markers as well as some more general clinical features. A detailed description of this database and reasoning behind the selection criteria for including recordings in the database can be found in (Chudáček et al., 2014).

### 2.2 Empirical dynamic modelling

Empirical dynamic modeling (EDM) is an emerging non-parametric framework for modeling non-linear dynamic systems (Chang et al., 2017). It is based on the mathematical theory of reconstructing attractor manifolds from time series data. Unlike models based on hypothesized parametric equations or known physical laws that describe simple idealized situations, empirical models infer patterns and associations from the data that are highly flexible and usually of great use in complex natural settings (Sugihara et al., 2020). The purpose of EDM is to infer the behavior of dynamic systems by reconstructing state space from time series data. This approach is based on mathematical theory developed initially in (Takens, 1981; Takens, 1985).

A direct application of EDM is causal discovery using reconstructed attractor manifolds, a method that is referred to as convergent cross mapping (CCM) and proposed in (Sugihara et al., 2012). From a dynamical system perspective, two time series are causally related if they are from the same dynamical system. In particular, let *M*_*x*_ and *M*_*y*_ denote the reconstructed attractor manifolds from time series *x*_*t*_ and *y*_*t*_, respectively. If *x*_*t*_ and *y*_*t*_ belong to the same dynamical system, *M*_*x*_ and *M*_*y*_ are topologically similar because they are embeddings of the (latent) attractor M of the system, and the signature of the causing series is encoded in the observed samples of the caused series (Wismüller et al., 2014; Schiecke et al., 2015).

### 2.3 Gaussian processes

A GP is a stochastic process with every finite set of random variables having a multivariate normal distribution (Rasmussen and Williams, 2006). A GP extends a multivariate Gaussian distribution to infinite dimensionality. Therefore, a GP can be seen as a distribution of a real-valued function *f*(**x**) in which **x** denotes the input and usually is a vector. The infinite dimensionality is actually easy to work with, given the marginalization property of multivariate Gaussian distributions. Further, latent functions can be conveniently marginalized out when computing model evidence.

A GP is characterized by its mean function *m*(**x**) and covariance function *k*_*f*_(**x**_*i*_, **x**_*i*_), which are defined by m(x)=E[f(x)] , and $k_{f}\left(x_{i},x_{j}\right)=E\left[\left(f\left(x_{i}\right)-m\left(x_{i}\right)\right)\left(f\left(x_{j}\right)-m\left(x_{j}\right)\right)\right]$ . To reduce the number of hyperparameters, in practice, a GP is assumed to be zero mean, that is, *m*(**x**) = 0 for every **x**. Furthermore, to preserve the tractability of the marginal likelihood, additive white Gaussian noise is usually adopted for modeling the observation noise, i.e., we write

$$y=y\left(x\right)=f\left(x\right)+\epsilon ,$$ (1)

where $\epsilon ∼N\left(0,\sigma_{\epsilon }^{2}\right)$ is additive white Gaussian noise.

The covariance matrix **K**_*ff*_ for training data can be obtained by evaluating the covariance function on **X**, i.e., **K**_*ff*_ = **k**_*f*_(**X**, **X**), where $X={\left\{x_{i}\right\}}_{i=1}^{N}$ denotes the collection of all training inputs. Then the likelihood of **f(X)** is given by

$$p\left(y|f\right)=N\left(y;f,\sigma_{\epsilon }^{2}I\right),$$ (2)

and the prior probability density function of **f**(**X**), which is specified by a GP, can be written as

$$p\left(f|X,\theta \right)=N\left(f;0,K_{ff}\right),$$ (3)

where ***θ*** denotes the set of hyperparameters used for modeling the covariance function.

Training refers to learning the model parameters, which include the hyperparameters ***θ*** and the variance of the observation noise $\sigma_{\epsilon }^{2}$ from the training data. These parameters are learned by maximizing the marginal likelihood. In the GP regression setting, the marginal likelihood can be obtained by marginalizing *f*, and the logarithm of the marginal likelihood defined by

$$\begin{array}{cc} \log p\left(y|X,\theta \right) & =\log N\left(y;0,\;K_{ff}+\sigma_{\epsilon }^{2}I\right)\\ & =\log N\left(y;0,\;K\right)\\ & =-\frac{1}{2}y^{T}K^{-1}y-\frac{1}{2}\log|K|-\frac{n}{2}\log2\pi \end{array}$$ (4)

can be used as an objective function for finding the.

We note that the last three terms in Eq. 4) have interpretations. The first one, $-\frac{1}{2}y^{T}K^{-1}y$ , is known as data-fit, which is the only term that involves the observations **y**. This term measures how well the model explains the data. The second term, $-\frac{1}{2}\log|K|$ , is known as penalty for the model complexity, and it depends on both the covariance function and the inputs. The third term, $-\frac{n}{2}\log2\pi$ , does not depend on the covariance matrix and the observations, and therefore, it is a normalization constant. The trade-off between data-fit and model complexity is automatic, which means that the tendency of the log marginal likelihood to favor more complex models is counterbalanced by the penalty for additional complexity. In other words, more complex models would have a better fit (a smaller value for **y** ^*T*^ **K** ^−1^ **y**) but a larger value of |**K**| than simpler models. This capability of the objective function (i.e., the log-likelihood) of making the GP robust to over-fitting reflects the principle of Occam’s razor. The hyper-parameters can be tuned by adopting a gradient-based optimizer.

For a test input X_*_, the predictive distribution of the test output, *p*(f_*_|X_*_, **X**, ***θ***), will be a Gaussian distribution with a mean and covariance given by

$$E\left(f_{*}\right)=\left[K_{f}\left(X_{*},X\right)\right]K^{-1}y,$$ (5)

$$cov\left(f_{*}\right)=K_{f}\left(X_{*},X_{*}\right)-\left[K_{f}\left(X_{*},X\right)\right]K^{-1}{\left[K_{f}\left(X_{*},X\right)\right]}^{⊤}.$$ (6)

We should note that the prediction is provided by way of a predictive distribution instead of a simple point estimate, which is preferable in many situations, particularly, in decision making. Since the mode of a Gaussian distribution is the same as its expectation, the mean of a predictive distribution, i.e., $E\left(f_{*}\right)$ is also the maximum *a posteriori* (MAP) estimate of **f**.

The covariance function transforms distance or similarity between inputs to covariance between outputs, and therefore, the design of the covariance function is critical in modeling. Perhaps the most widely adopted covariance function is the squared exponential or radial basis function (RBF). Its general form is given by

$$k\left(x,x^{\prime }\right)=\sigma_{f}^{2}\exp\left(-\frac{1}{2}\overset{Q}{\underset{q=1}{\sum }}\frac{1}{\ell_{q}}{\left(x_{q}-x_{q}^{\prime }\right)}^{2}\right),$$ (7)

where the characteristic length-scale *ℓ*_*q*_ > 0 and the signal variance $\sigma_{f}^{2}$ are its hyperparameters. These parameters are interpretable; for instance, $\sigma_{f}^{2}$ measures the strength or variability of the corresponding function, whereas *ℓ*_*q*_ controls the model complexity in the *q*th dimension since the input distance will be scaled by *ℓ*. Therefore, if *ℓ* is small, a small change in the input distance will cause a large change in the covariance of the outputs, and *vice versa*. Equivalently, one can define a relevance weight by $r_{q}=\frac{1}{\ell_{q}}$ to measure the importance or relevance of that dimension in the modeling. Since *r*_*q*_ is automatically learned from training data, this is known as automatic relevance determination (ARD) (Rasmussen and Williams, 2006). In supervised learning, ARD can be applied for automatic feature selection, and in unsupervised learning it can be utilized for automatic dimensionality reduction (Lawrence, 2004; Damianou et al., 2012; 2016). Another popular family of covariance functions is the Matérn class of functions. The parameter that defines them is denoted as *ν*. When *ν* is half integer, it can be shown that the Matérn covariance functions become simply a product of an exponential and a polynomial. Its one dimensional form corresponding to *ν* = 3/2 is as follow:

$$k_{\nu =3/2}\left(d\right)=\sigma_{f}^{2}\left(1+\sqrt{3}d/\ell \right)\exp\left(-\sqrt{3}d/\ell \right),$$ (8)

where *d* is the distance between *x*_*i*_ and *x*_*j*_. Unlike the RBF covariance function, the Matérn covariance functions generally models rough processes. More information on the designing of covariance functions can be found in (Rasmussen and Williams, 2006).

## 3 Model description

### 3.1 Taken’s theorem

We reiterate that the core of EDM is state space reconstruction based on Takens’ theorem, which is stated as follows:

Theorem 1 (Takens’ theorem). *Let* M *be a compact manifold of (integer) dimension* *d* *. Then for generic pairs* $\left(\phi ,y\right)$ *, where*

• ϕ:M→M *is a* C^2^ *-diffeomorphism of* M *in itself,*
• y:M→R *is a* C^2^ *-differentiable function,*

*the map* $\Phi_{\left(\phi ,y\right)}:M\to R^{2d+1}$ *given by*

$$\Phi_{\left(\phi ,y\right)}\left(x\right):=\left(y\left(x\right),y\left(\phi \left(x\right)\right),y\left(\phi^{2}\left(x\right)\right),\ldots ,y\left(\phi^{2d}\left(x\right)\right)\right)$$

*is an embedding of* M *in* $R^{2d+1}$ *.*

In the literature of EDM, the most popular choice of *ϕ* is delay embedding, i.e., delay by a constant *τ*. Takens’ theorem provides a theoretical foundation that under some mild conditions, we can reconstruct M by using, e.g., delay embedding of dimension *E* = 2*d* + 1 from a single observation variable of the system. Specifically, given a time series *x*(*t*), for each time instant *t*, an *E* dimensional vector **m**_*x*_(*t*) = [*x*(*t*),*x*(*t*−*τ*),…,*x*(*t*−(*E*−1)*τ*)]^*⊤*^ is constructed. Then each $m_{x}\in R^{E}$ is collected as a row in a matrix $M_{x}^{\text{DE}}\in R^{N\times E}$ where *N* is the number of **m**_*x*_ vectors constructed from *x*(*t*). Essentially, $M_{x}^{\text{DE}}$ is a reconstructed attractor manifold with *E*-dimensional delay embedding from *x*(*t*).

Despite the sound theoretical foundation established by Takens’ theorem, some details need to be addressed before applying Takens’ theorem. In most situations we do not have perfect knowledge regarding the underlying dynamical system that generates the observations, and therefore, the true dimensionality of attractor manifold *d* remains latent to us. Although Takens’ theorem states that *E* = 2*d* + 1 should be sufficient, *E* = 2*d* + 1 is essentially latent as well. If the embedding dimension *E* is too small, then the reconstructed states can overlap and can appear to be the same even though they actually correspond to different states. On the other hand, if *E* is too large, the subsequent analysis will suffer from the curse of dimensionality (Sugihara et al., 2012).

In the literature of EDM, *E* is either selected empirically based on domain knowledge or by grid-search type methods. For instance, in (Ye et al., 2015) the embedding dimension is selected using the false nearest neighbours (Kennel et al., 1992) and grid searching from *E* = 2 to *E* = 8. Similarly, in (Sugihara et al., 2012) and (Sugihara et al., 2020), *E* is determined by prediction performance and grid searching from *E* = 2 to *E* = 10. In order to properly measure the prediction performance, performance metrics are averaged over a large number of randomly initialized experiments for each value of *E* in the gird, and this can be computationally expensive. As mentioned in (Sugihara et al., 2012; 2020), the optimal *E* that achieves the best prediction performance in the grid search does not necessarily correspond to the dimensionality of the original dynamical system.

Furthermore, in theory, the choice of *τ* can be arbitrary because it is not specified in Takens’ theorem. However, the choice of *τ* will indeed affect the quality of attractor manifold reconstruction. If *τ* is too small, each dimension will be strongly correlated. On the other hand if *τ* is too large, we will lose information about the underlying dynamical system. In practice, the value of *τ* is often selected using a mutual information based approach (Fraser and Swinney, 1986), and it also relies on a grid search. Finally, since observational noise is not modeled in delay embedding, the reconstruction result is not robust to observational noise. Clearly, this undermines its applicability to real-world data.

### 3.2 Empirical dynamic modeling with GP

Recently it has been shown that the dynamics of processes can be learned with continuous time latent processes that are parametrized by neural ordinary differential equations (Chen et al., 2018). However, learning the parameters in the neural networks requires a large amount of training data, which can be difficult in applications where observations are expensive to collect. Instead, EDM and GPs can exploit the widely adopted delay embedding and estimate a latent representation of the state space. Conceptually, EDM with a GP combine the convenience of delay embedding and power of representation learning.

In the initial reconstruction step using delay embedding, instead of performing grid search for selecting *E* and *τ*, we directly assign a relatively large *E*, e.g., *E* = 10 or *E* = 20, to fulfill the requirement of Taken’s theorem. To preserve the dynamical information, we choose *τ* = 1, i.e., a delay by one sample, which is the smallest delay. We denote this initial reconstruction as *M* ^init^. That is, given a time series *x*_*t*_, an *E*-dimensional vector $m_{x}^{\text{init}}\left(t\right)={\left[x\left(t\right),x\left(t-1\right),\ldots ,x\left(t-\left(E-1\right)\right)\right]}^{⊤}$ can be found on *M* ^init^ for time instance *t*. We should note that $M^{\text{init}}\in R^{N\times E}$ is not only of high dimensionality but also with high correlation between the different dimensions.

Then we apply a GP latent variable model (GPLVM) (Titsias and Lawrence, 2010) to infer a lower dimensional latent representation of *M* ^init^, denoted as *M* ^GP^. The generative model is as follows:

$$M^{\text{init}}=f\left(M^{\text{GP}}\right)+\epsilon ,$$ (9)

where $\epsilon \in R^{N\times \hat{d}}$ is a matrix whose rows are zero mean Gaussian with covariance $\sigma_{\epsilon }^{2}I$ . We initialize each dimension in **f** as an independent draw from a GP where the covariance function is a multi-dimensional radial basis function (RBF) conversion function in Eq. 7). Conceptually, we remove the redundancy of the dimensions in *M* ^init^ with the GPLVM.

The learning requires maximizing the marginal likelihood given by:

$$p\left(M^{\text{init}}\right)=\int p\left(M^{\text{init}}|M^{\text{GP}}\right)p\left(M^{\text{GP}}\right)dM^{\text{GP}}.$$ (10)

Unlike the GP regression framework, this marginal likelihood is intractable because *M* ^GP^ and *M* ^init^ are related by the covariance function in a highly non-linear manner, and in general, non-linear mapping will not preserve Gaussianity. This is handled in (Titsias and Lawrence, 2010) by employing variational inference and approximating the true posterior *p*(*M* ^GP^∣*M* ^init^) by a Gaussian variational distribution *q*(*M* ^GP^). From this distribution, we obtain a tractable lower bound on the marginal likelihood, which is used for learning.

The ARD weights embedded in the covariance function enable us to estimate the dimensionality of the underlying latent attractor manifold, i.e., *d*, since irrelevant dimensions will be assigned weights close to zero. Instead of initializing $\hat{d}<E$ , we initialize $\hat{d}$ to be equal to *E*. If the non-linear mappings governed by GP fail to remove redundancy in *M* ^init^, e.g., *E* is not sufficiently large in the first place, we do not enforce compression. Finally, we use the mean of *q*(*M* ^GP^) as the reconstructed attractor manifold after removing its irrelevant dimensions, and the uncertainty of learning is captured in the covariance of *q*(*M* ^GP^).

### 3.3 A toy example

**FIGURE 2:**
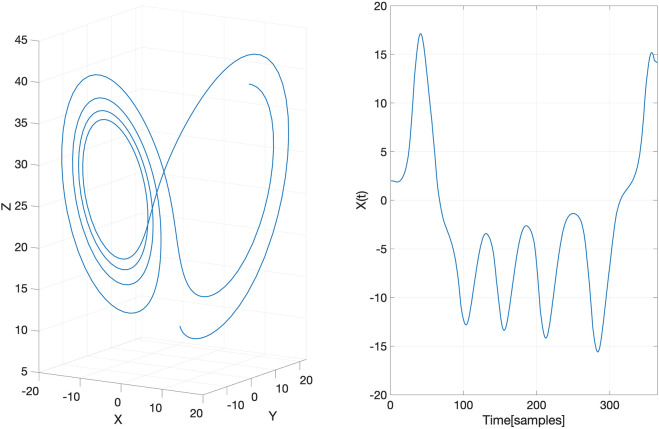
The underlying latent Lorenz attractor (left) generated in the toy example and its projection on the *X*-axis (right).

We demonstrate the aforementioned approach of EDM using the Lorenz system (Tabor and Weiss, 1981; Emanuel, 1994) which is non-linear, non-periodic, three-dimensional and deterministic. It has been well-studied for having chaotic solutions for certain parameter values and initial conditions. To demonstrate that our GP-based method can provide reliable reconstruction of an attractor manifold, we generated a Lorenz attractor (a set of chaotic solutions of the Lorenz system) M with the following equations:

$$\begin{array}{cc} & dx/dt=a\left(y-x\right),\\ & dy/dt=x\left(c-z\right)-y,\\ & dz/dt=xy-bz, \end{array}$$ (11)

and a classic set of parameter values *a* = 10, $b=\frac{8}{3}$ , and *c* = 28. Further, we assumed that only the projection on the *X*-axis, denoted as *X*(*t*), was observed. The generated Lorenz attractor and its projection *X*(*t*) are plotted in Figure 2.

**FIGURE 3:**
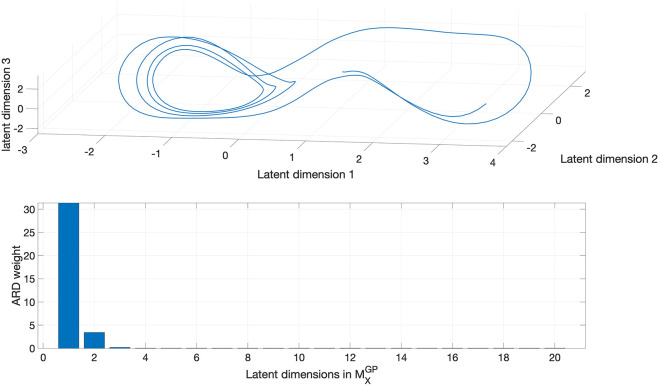
The reconstructed Lorenz attractor from *X*(*t*) using GP-based approach (top) and ARD weights of learned latent dimensions (bottom). The number of dimensions with non-zero ARD weights is three, which is the true dimension of the Lorenz attractor. The GP-based approach is capable of learning the dimension of the latent attractor from data in an automated manner.

Then we reconstructed the attractor manifold from *X*(*t*) with our GP-based method. The reconstruction results are shown in Figure 3, where we can see that the $M_{X}^{\text{GP}}$ is topologically similar to the underlying latent Lorenz attractor. Moreover, the true dimension of the Lorenz system, which is 3, was correctly revealed by the ARD weights although we initialized *Q* = 20. It can be shown that the GP-based reconstruction is also robust to observation noise. More details on this can be found in (Feng et al., 2020b).

## 4 Application I: Causal discovery between FHR and UA

As mentioned in Section 2.2, an immediate application of EDM is CCM for causal discovery from time series data. The basic idea of CCM is to measure the extent to which the historical record of one variable can reliably estimate states of the other variable using simplex projections (Sugihara and May, 1990). Essentially, CCM tests whether or not a neighborhood defined on *M*_*x*_ is preserved on *M*_*y*_, and *vice versa*, i.e., for causal discovery it looks for a signature of cause in the history of the effect. More details about the CCM framework can be found in (Sugihara et al., 2012).

**FIGURE 4:**
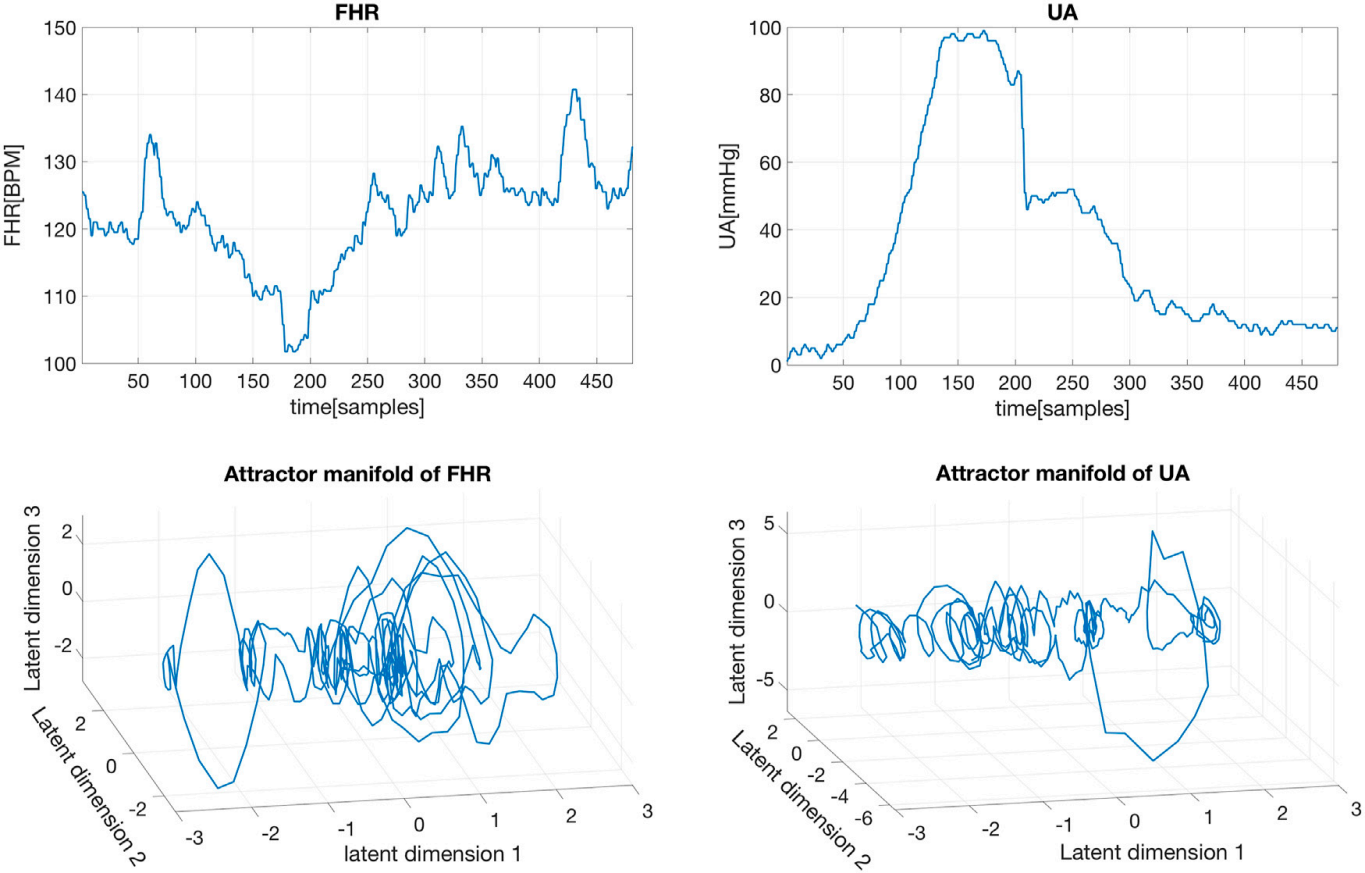
Raw FHR and UA segments (top), and their corresponding reconstructed attractor manifolds with the GP-based method (bottom).

Although in the literature on obstetrics and gynecology it has been well recognized that changes on UA can cause changes in FHR (Spinnewijn et al., 1996; Nageotte, 2015; Sletten et al., 2016), we confirmed this conclusion by testing causality between the FHR and UA signals within the CCM framework. Specifically, we first reconstructed the attractor manifolds from the FHR and UA signals using the GP-based EDM. For instance, a short segment of FHR and UA signals and their corresponding reconstructed attractor manifolds are shown in Figure 4. Then we carried out the simplex projection algorithm using GP regression, similar to the original simplex project in (Sugihara and May, 1990). However, unlike the original simplex projection, the GP-based simplex projection is more robust to noise since the observation noise is considered explicitly in the generative process as shown in Eq. 1). Considering that the causality between FHR and UA signals is well recognized and due to limited space, the CCM framework and GP-based simplex projection are not described further here. More details about this can be found in (Feng et al., 2020a). It is worth noting that the developed framework in (Feng et al., 2020a) can readily be applied to causal discovery in other communities.

## 5 Application II: Characterizing interactions between FHR and UA signals

In the evaluation performed by obstetricians, the FHR signal is assessed with reference to its corresponding UA signal. For instance, widely adopted FHR patterns are the early deceleration, which is defined as a symmetrical decrease and return back to the previous level and similarly, the late deceleration is defined as a visually apparent and gradual decrease in the FHR typically following the uterine contraction. In the literature of automated analysis of FHR, the interaction between FHR and UA is usually not considered. In this section, we show that the GP-based EDM can be applied to characterizing the interaction between FHR and UA signals.

We adopted the same open access database, and we used the first 30 min of FHR and UA signals in this experiment because when approaching the end of labor, the signal quality of both FHR and UA recordings deteriorates noticeably (Spilka, 2013). We used the preprocessing algorithm described in (Spilka, 2013). One CTG recording was excluded from our experiment because its UA recording is empty. Then for each CTG recording, we reconstructed the attractor manifolds of the FHR and UA signals using the GP-based method.

We used the Hausdorff distance to measure the distance or similarity between the reconstructed attractor manifolds (of the FHR and UA signals). In other words, for each CTG recording, we utilized the Hausdorff distance between $M_{\text{FHR}}^{\text{GP}}$ and $M_{\text{UA}}^{\text{GP}}$ to characterize the interaction between the FHR and UA signals. The Hausdorff distance measures the degree of mismatch between two set of points *A* and *B* (Shapiro and Blaschko, 2004), and it is defined as follows:

$$H\left(A,B\right)=\max\left\{\underset{a\in A}{\sup}d\left(a,B\right),\underset{b\in B}{\sup}d\left(A,b\right)\right\},$$ (12)

where

$$d\left(a,B\right)=\underset{b\in B}{\inf}d\left(a,b\right)$$ (13)

and *d*(*a*, *b*) represents the Euclidean distance between point *a* and point *b*.

In computerized analysis of CTG, the gold standard for labeling FHR tracings has been the umbilical arterial pH value of the fetus at birth (Armstrong and Stenson, 2007), although the choice of a cutoff value to determine “acidosis” has not been universally accepted (Georgieva et al., 2013; Abry et al., 2018). The popular FHR features generally are not well correlated with the pH value (the absolute value of the Pearson correlation coefficient between a feature and pH value is close to zero) (Fulcher et al., 2014). Therefore, the Pearson correlation coefficient with pH is an important well-adopted metric when proposing and selecting FHR features, and we adopted it for evaluating $H\left(M_{\text{FHR}}^{\text{GP}},M_{\text{UA}}^{\text{GP}}\right)$ .

For comparison purposes, we selected popular time domain features including the short term variability (STV) and the long term variability (LTV) (Gonçalves et al., 2006) as well as frequency domain features proposed in (Signorini et al., 2003). Further, we used non-linear domain features including the approximate entropy and the sample entropy (Delgado-Bonal and Marshak, 2019). The frequency domain features proposed in (Signorini et al., 2003) were the energies in four frequency bands: very low frequency (VLF): 0–.06 Hz, low frequency (LF): .06–.3 Hz, medium frequency (MF): .3–1 Hz and high frequency (LF): 1–2 Hz; and the ratio of energies defined by LF/(MF + HF).

**FIGURE 5:**
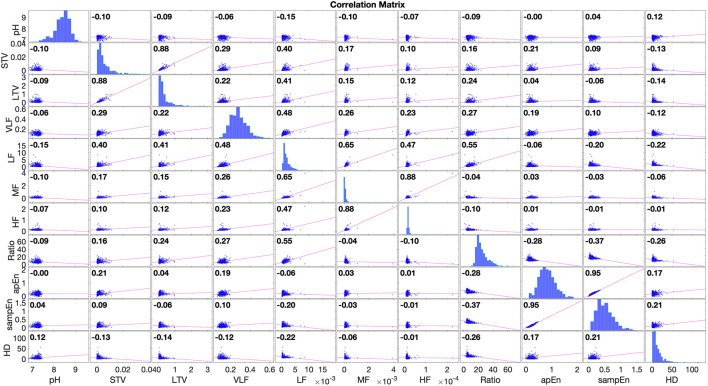
The correlation matrix of features and umbilical artery pH, computed with 552 CTG recordings in an open access CTG database described in Chudáček et al. (2014). The correlation coefficient between $H\left(M_{\text{FHR}}^{\text{GP}},M_{\text{UA}}^{\text{GP}}\right)$ , denoted as HD, and pH is comparable with popular features such as STV, LTV and LF. Meanwhile $H\left(M_{\text{FHR}}^{\text{GP}},M_{\text{UA}}^{\text{GP}}\right)$ is not highly correlated with other FHR features.

The correlation matrix of the features and the umbilical artery pH is illustrated in Figure 5. The correlation coefficient between the umbilical cord artery pH value and $H\left(M_{\text{FHR}}^{\text{GP}},M_{\text{UA}}^{\text{GP}}\right)$ is −.12, which is comparable with that of the popular FHR features such as STV, LTV and LF. Meanwhile, $H\left(M_{\text{FHR}}^{\text{GP}},M_{\text{UA}}^{\text{GP}}\right)$ is not highly correlated with these popular FHR features, the highest correlation coefficient between $H\left(M_{\text{FHR}}^{\text{GP}},M_{\text{UA}}^{\text{GP}}\right)$ and other FHR features being −.26. This indicates that $H\left(M_{\text{FHR}}^{\text{GP}},M_{\text{UA}}^{\text{GP}}\right)$ is not only interpretable but also able to provide additional information on the umbilical artery pH.

Despite the popularity of labeling, CTG recordings using umbilical artery pH in computerized analysis, a few caveats should be noted. One is that, there may be an implicit bias in using pH values as a label because in many hospitals this value is acquired only after a pregnancy is declared or suspected to be of “high risk.” Further, the umbilical artery pH value can change over time. This indicates that the arterial pH has intrinsic variance and its value may be affected by factors such as duration of labor, cord blood sampling technique and total time elapsed between delivery of the fetus and acquisition of the umbilical cord blood sample. Finally, we recall that there are commonly adopted clinical evaluations of fetal wellbeing post-delivery, e.g., *via* Apgar scores, but they have inter-observer and intra-observer variability.

## 6 Application III: Estimating missing samples in FHR

In practice, many factors may affect the quality of the CTG signals in their acquisition, for instance, intra-recording displacement of the ultrasound probe, fetal or maternal movement, and technician’s expertise and experience (Hamelmann et al., 2016). A particular challenge in the analysis for CTG recordings is the large number of missing samples in FHR. For example, when using Doppler-based FHR measurements, the percentage of missing samples can vary from 0%–40% (Oikonomou et al., 2013). Such signal loss episodes have various causes, e.g., fetal or maternal movement, and misplaced transducer. The missing samples introduce variability and uncertainty to the extracted features. This not only indicates the necessity of adopting a probabilistic framework where we can properly express the uncertainties, but also suggests the need for appropriate treatment of the missing samples. Because many computerized approaches rely on features extracted from FHR recordings, these missing samples can cause serious problems if they are not properly addressed. In (Spilka et al., 2012), the authors investigated the stability of several commonly used FHR features when there were missing samples, and their experimental results showed that the feature values changed dramatically with the increase of missing samples. In automated FHR analysis, small segments of missing samples are interpolated with linear or cubic spline interpolation, and longer consecutive segments are often entirely removed (Sprott and Sprott, 2003).

In this application, we take causal relationship between FHR and UA into consideration when estimating missing samples in FHR recordings. Particularly, for reliable recovery of missing samples in FHR, we propose a GP-based method using GP regression which is capable of incorporating UA signals for the estimate of missing FHR samples automatically (Feng et al., 2017). We model the observed value of the *i*th sample *y*_*i*_ in a FHR segment as a function of the time index *i* and its synchronized UA sample, *u*_*i*_, with additive Gaussian white noise, i.e.,

$$y_{i}=y\left(x_{i}\right)=f\left(x_{i}\right)+\epsilon ,$$ (14)

where $x_{i}={\left[i,u_{i}\right]}^{\prime }$ is a 2-D vector, *f*(**x**_*i*_) is a latent variable, and $\epsilon ∼N\left(0,\sigma^{2}\right)$ is Gaussian white noise.

We designed the covariance function for this task as a sum of an RBF covariance function (for capturing slow varying components), a Matérn covariance function (*ν* = 3/2, for capturing rapid varying components), and a linear covariance function (for capturing linearity). Its specific form is as follows:

$$\begin{array}{ll} k_{f}\left(x_{i},x_{j}\right)= & {\alpha_{1}}^{2}\left[1+\sqrt{3}{\left[{\left(x_{i}-x_{j}\right)}^{\prime }\Lambda_{1}\left(x_{i}-x_{j}\right)\right]}^{\frac{1}{2}}\right]\\ & \times \exp\left[-\sqrt{3}{\left[{\left(x_{i}-x_{j}\right)}^{\prime }\Lambda_{1}\left(x_{i}-x_{j}\right)\right]}^{\frac{1}{2}}\right]\\ & +{\alpha_{2}}^{2}\exp\left[-\frac{1}{2}{\left[{\left(x_{i}-x_{j}\right)}^{\prime }\Lambda_{2}\left(x_{i}-x_{j}\right)\right]}^{\frac{1}{2}}\right]\\ & +\left[{\left(x_{i}\right)}^{\prime }\Lambda_{3}\left(x_{j}\right)\right], \end{array}$$ (15)

where $\Lambda_{1}=\left(\begin{array}{cc} \beta_{1} & 0\\ 0 & \beta_{2} \end{array}\right)$ , $\Lambda_{2}=\left(\begin{array}{cc} \beta_{3} & 0\\ 0 & \beta_{4} \end{array}\right)$ and $\Lambda_{3}=\left(\begin{array}{cc} \beta_{5} & 0\\ 0 & \beta_{6} \end{array}\right)$ .

**FIGURE 6:**
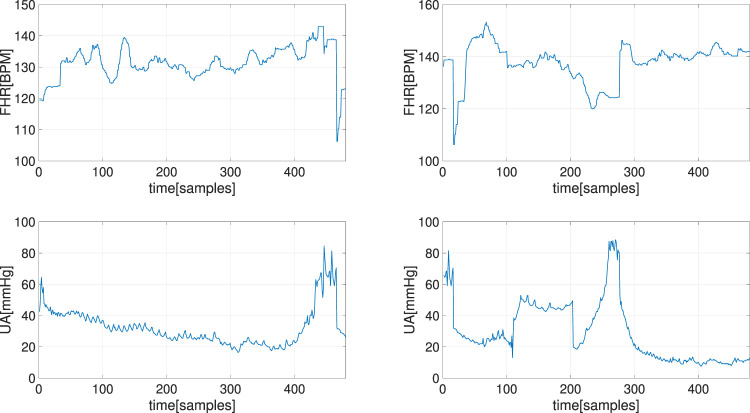
The two CTG segments, i.e., FHR (top) and UA (bottom) recordings, used for estimating of random missing samples in FHR.

**FIGURE 7:**
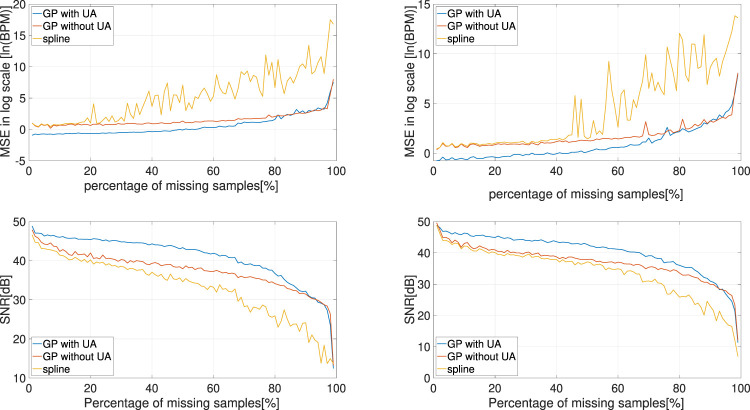
Experimental results of estimating random missing samples in FHR: The experiment results for the first CTG segment (left), and the experiment results for the second CTG segment (right). The MSE (upper plot, in logarithmic scale) and SNR (bottom plot) of each method under different percentages of missing samples are presented, averaged over 100 experiments.

We tested this approach on two raw CTG segments of length 2 min that are plotted in Figure 6. We evaluated the performance by investigating the recovery performance with respect to different percentages of missing samples, which ranged from 1% to 99% with a step size of 1% on both CTG segments. For each percentage, the experiment was repeated 100 times and the performance metrics were averaged over 100 results. To demonstrate the contribution of the UA signal, the experiments were repeated using a similar GP model but with the UA samples excluded from the input of the latent function, i.e., *u*_*i*_ was taken out from $x_{i}={\left[i,u_{i}\right]}^{\prime }$ . Cubic spline interpolation was also adopted for benchmarking since it is widely applied in practice. The recovery performance was measured by the mean squared error (MSE) in logarithmic scale and the signal-to-noise ratio (SNR), which were defined by

$$\text{Log MSE}=\log_{e}\left(\frac{{\left\|s-\hat{s}\right\|}^{2}}{N}\right),$$ (16)

and

$$\text{SNR}=10\log_{10}\left(\frac{{\left\|s\right\|}^{2}}{{\left\|s-\hat{s}\right\|}^{2}}\right),$$ (17)

where *N* is the number of missing samples, **s** is the ground truth and $\hat{s}$ is the reconstructed signal. The experimental results, for both CTG segments in Figure 6, are shown in Figure 7, and they clearly show that the GP-based method, when incorporated the UA signals, provides the best performance. For example, even when the percentage of missing samples was more than 50%, the MSE of our approach was still around one beat per minute in both cases.

A more challenging situation in estimating the missing samples in FHR recordings is that when the missing samples are consecutive (i.e., they appear in bursts) and their corresponding UA samples are also missing. In (Feng et al., 2021), we proposed to utilize the attractor manifold of FHR signal learned by GP-based EDM for this task, because it enables us to identify similarity in terms of the state of system. Particularly, in the generative process, on top of the GP-based EDM, we explicitly correlated points on the attractor manifold in time by modeling $M_{\text{FHR}}^{\text{init}}$ as the output of a dynamically constrained deep GP.

## 7 Conclusion

In this paper, we present a GP-based EDM for state space reconstruction from time series data, which is able to estimate the attractor manifold within probabilistic framework. The dimensionality of the attractor manifold is also simultaneously learned from observations, which is more principled comparing to the classical EDM with direct delay embedding where the parameters are selected using grid search-based methods. Furthermore, the learning is captured by the covariance of the variational distribution *q*(*M* ^GP^), which is important in many applications especially in decision making. Unlike the traditional EDM with direct delay embedding, the observation noise is explicitly modeled in the GP-based EDM, and as a result, the GP-based EDM is more robust to observation noise. Comparing with EDM using neural ODE, the GP-based EDM is data efficient because the number of model parameters are much less then that of the neural networks. Improving the state space reconstruction is beneficial for subsequent analysis of the FHR and UA signals. For instance, in CCM for casual discovery, the correspondence between the reconstructed attractor manifolds is utilized to detect causality.

It is well recognized, from clinical point of view, that changes in the UA signal can cause changes in the FHR signal. In computerized analysis of CTGs, we first confirmed this conclusion within the CCM framework by testing the correspondence between the reconstructed attractor manifolds of the FHR and UA signals. This is also the logic behind taking the UA signal into consideration in automated analysis of CTGs. The GP-based EDM enables us to compare the FHR and UA signals simultaneously from a dynamical system point of view. As a direct application of a GP-based EDM, we then used the Hausdorff distance between the reconstructed attractor manifolds of the FHR and UA signals, i.e., $H\left(M_{\text{FHR}}^{\text{GP}},M_{\text{UA}}^{\text{GP}}\right)$ , to characterize the interaction between the FHR and UA signals. We showed that $H\left(M_{\text{FHR}}^{\text{GP}},M_{\text{UA}}^{\text{GP}}\right)$ is able to provide additional information about the umbilical artery pH. Further, we addressed the problem of missing samples in the CTGs. The treatment of missing samples is often the very first step in preprocessing the FHR and UA signals, which in turn plays an important role in all of the downstream analysis of these signals. Utilizing causal relationship is more reliable and desirable in this task compared to correlation which can be spurious and inconsistent. As an indirect application of GP-based EDM, we also used the causal relationship between the FHR and UA signals for improved estimation of missing samples in the recordings.

## Data Availability

The original contributions presented in the study are included in the article, further inquiries can be directed to the corresponding authors.
